# The Allelic Variant A391T of Metal Ion Transporter ZIP8 (SLC39A8) Leads to Hypotension and Enhanced Insulin Resistance

**DOI:** 10.3389/fphys.2022.912277

**Published:** 2022-06-15

**Authors:** Sophia N. Verouti, Jonai Pujol-Giménez, Paola Bermudez-Lekerika, Laeticia Scherler, Rajesh Bhardwaj, Aurélien Thomas, Sébastien Lenglet, Mark Siegrist, Willy Hofstetter, Daniel G. Fuster, Matthias A. Hediger, Geneviève Escher, Bruno Vogt

**Affiliations:** ^1^ Department for BioMedical Research (DBMR), University of Bern, Bern, Switzerland; ^2^ Department of Nephrology and Hypertension, Inselspital, Bern University Hospital, University of Bern, Bern, Switzerland; ^3^ Signal Transduction Laboratory, National Institute of Environmental Health Sciences, NIH, Durham, NC, United States; ^4^ Forensic Toxicology and Chemistry Unit, CURML, Lausanne University Hospital, Geneva University, Geneva, Switzerland; ^5^ Faculty Unit of Toxicology, CURML, Faculty of Biology and Medicine, University of Lausanne, Lausanne, Switzerland

**Keywords:** ZIP8, blood pressure, glucose, SLC39A8, rs13107325, divalent metal ions, metal ion transporters

## Abstract

The metal ion transporter ZIP8 (SLC39A8) mediates cellular uptake of vital divalent metal ions. Genome-wide association studies (GWAS) showed that the single-nucleotide polymorphism (SNP) variant A391T (rs13107325) is associated with numerous human traits, including reduced arterial blood pressure, increased body mass index and hyperlipidemia. We analyzed *in vitro* the transport properties of mutant ZIP8 A391T and investigated *in vivo* in mice the physiological effects of this polymorphism. *In vitro*, the intrinsic transport properties of mutant ZIP8 were similar to those of wild type ZIP8, but cellular uptake of zinc, cadmium and iron was attenuated due to reduced ZIP8 plasma membrane expression. We then generated the ZIP8 A393T mice (ZIP8KI) that carry the corresponding polymorphism and characterized their phenotype. We observed lower protein expression in lung and kidney membrane extracts in ZIP8KI mice. The ZIP8KI mice exhibited striking changes in metal ion composition of the tissues, including cobalt, palladium, mercury and platinum. In agreement with GWAS, ZIP8KI mice showed reduced arterial blood pressure. Body weight and plasma lipid composition remained unchanged, although these features were reported to be increased in GWAS. ZIP8KI mice also exhibited remarkable insulin resistance and were protected from elevated blood glucose when challenged by dietary sucrose supplementation. We showed that increased hepatic insulin receptor expression and decreased ZnT8 (slc30a8) metal ion transporter mRNA expression are associated with this phenotypic change. In conclusion, our data reveal that ZIP8 plays an important role in blood pressure regulation and glucose homeostasis.

## Introduction

Divalent metal ions are of vital importance as they participate in many biochemical reactions. They are effective in low concentrations and their presence in body fluids must be tightly regulated, since both a deficiency and an excess can lead to severe illness or even death. SLC39A8 (ZIP8) acts like several other SLC39 (ZIP) family members as a transporter of different divalent metal ions such as Zn^2+^, Cd^2+^, Fe^2+^, Mn^2+^, Hg^2+^ and Co^2+^, with ZIP8 and ZIP14 showing a particular promiscuity toward metals ions (Nebert et al., 2012; Wang et al., 2012; Steimle et al., 2019). ZIP8-mediated metal transport has been proposed to be electroneutral, due to coupling to the co-transport of HCO3^−^ (He et al., 2006; Nebert et al., 2012). Most abundant ZIP8 expression was detected in lung and placenta followed by salivary gland and thymus (Wang et al., 2012).

In humans, ZIP8 loss of function mutation is linked to trace mineral deficiencies and inherited glycosylation disorders (Park et al., 2015). Variants in SLC39A8 impair the function of the manganese-dependent enzyme like β-1,4-galactosyltransferase (Park et al., 2015). The SNP rs13107325 with an allelic frequency of 5%–10% in the general population is located on exon 8, with the major allele having a cytosine (C) and the minor allele a thymidine (T) at the first position of the codon for residue 391. In several genome wide association studies (GWAS), rs13107325, was associated with schizophrenia, reduced arterial blood pressure and increased body mass index and HDL-cholesterol (HDL-C) (Costas, 2018; Parisinos et al., 2020). It was first proposed that rs13107325 may not affect gene transcription (Zhang et al., 2016) and, later on, it was suggested that it could compromise protein function (Zhang et al., 2016). However, the precise molecular basis for these traits due to rs13107325 remains elusive.

Few mouse models have been created to study the role of ZIP8 in physiology (Gálvez-Peralta et al., 2012; Kim et al., 2014; Lin et al., 2017; Sunuwar et al., 2020). In the present study, we generated ZIP8KI mice carrying the human variant Thr391 to examine the characteristic phenotypes that were identified in the GWAS (Costas, 2018; Parisinos et al., 2020). We also assessed the implication this variant in other physiological processes that have not yet been considered, such as glucose metabolism. Additionally, an evaluation of the levels of divalent metals in serum and tissue and a deep *in vitro* characterization of the ZIP8 Thr391 variant were carried out to better understand the effect of the polymorphism on transporter activity.

## Methods

### Homology Modelling

The UniProt sequence Q91W10 (UniProt Consortium, 2018) of mouse ZIP8 was submitted to the SWISS-MODEL homology modelling server (Waterhouse et al., 2018). Briefly, the SWISS-MODEL template library was searched with BLAST (Camacho et al., 2009) and HHBlits (Remmert et al., 2011) for evolutionary related structures matching the submitted sequence. Possible templates (2080) were selected for model building. Models were generated using the ProMod3 3.0.0 modelling engine. Global and per-residue quality was assessed using the Qmean scoring function. The best model showing best scores and structure coverage was selected. Models were built based on the inward-open *Bordetella Bronchiseptica* BbZIP X-Ray crystal structure (PDB ID:6PGI) (Zhang et al., 2017). The selected model was submitted to the Orientation of Proteins in Membranes (OPM) database (Lomize et al., 2006), to generate the final membrane-embedded model for mouse ZIP8. Images were generated with the UCSF Chimera 1.4 software (Pettersen et al., 2004).

### Generation and Sub-Cloning of SLC39A8 Mutant

The human SLC39A8 (ZIP8) ORF (NCBI Reference Sequence: NM_022154.5) containing a C-terminal TurboGFP tag, was cloned into the pCMV6-AC-GFP vector (OriGene #RG204200). After PCR amplification, human SLC39A8 was subcloned into the pIRES2 DsRed-Express2 vector (Takara Clontech) without the GFP tag. The Ala391Thr mutant was generated by site-directed mutagenesis using the polymerase chain reaction (PCR) based method as previously described (Manatschal et al., 2019). 5′-GGT GGG CAA CAA TTT CAC TCC AAA TAT TAT ATT TGC-3′ primer was used for mutagenesis using human SLC39A8 pIRES2 DsRed-Express2 construct as a template for PCR.

5′-GCA GAG CTG GTT TAG TGA ACC G-3′ forward and 5′-ATA TGA ATT CTT ACG CAT AGT CTG GGA CGT CGT ACG GAT ATC CCT CCA ATT CGA TTT CTC CT GC-3′ reverse primers were used for PCR amplification of human SLC39A8 WT and A391T mutant with C-terminal HA tag overhangs using SLC39A8 WT and A391T pIRES2 DsRed-Express2 expression constructs, respectively as templates. The WT and A391T PCR products containing C-terminal HA tags were digested with XhoI and EcoRI-HF restriction enzymes (New England Biolabs) and ligated with purified pIRES2 DsRed-Express2 vector digested with the same enzymes. The final constructs are human SLC39A8-HA pIRES2 DsRed-Express2 and SLC39A8-A391T-HA pIRES2 DsRed-Express2.

### Cell Culture

HEK293T cells (ATCC) were cultured in Dubelcco’s modified Eagle medium (DMEM) supplemented with 10% fetal bovine serum (FBS), 10 mM HEPES, 1 mM sodium pyruvate and 100 μM minimal essential medium (MEM) nonessential amino acids. Cells were maintained at 37°C in a humidified 5% CO_2_ incubator. Cells were seeded into poly-D-lysine coated, clear bottom, 96-well plates at a density of 250,000 cells/ml or into standard cell culture 6-well plates at a density of 1 million cells/well. Transfection was performed with the indicated DNA plasmids using the Lipofectamine 2000 (Life Technologies) as indicated in the manufacturer’s protocol. Experiments were performed 24 h after transfection.

### Intracellular Zinc Accumulation Measurements by Fluorescence Microscopy

Cells were grown in 96-well plates and incubated with 50 μl of Fluo-4 NW dye (ThermoFisher) in calcium free uptake buffer (117 mM NaCl, 4.8 mM KCl, 1 mM MgCl_2_, 10 mM glucose, 10 mM HEPES, pH 7.4) for 45 min at 37°C. Then, 50 μl of the indicated concentration of ZnCl_2_ were added for additional 15 min at 37°C. After the incubation period had elapsed, fluorescence imaging was performed on a IMIC digital microscope (FEI, Type 4001) using the Polychrome V light source, an Orca-R2 camera controller from Hamamatsu (C10600) and Live Acquisition software (FEI, version 2.6.0.14). To quantify the fluorescence intensity, the average optical density of the images, previously corrected to the background non-specific signal determined in non-transfected cells, was determined using the ImageJ software (National Institutes of Health) (Schneider et al., 2012). The experiment was performed the indicated number of independent times, each experimental condition was measured in 6–12 wells per plate, and a single picture was taken and quantified from each well. Pictures showing significantly different number of cells or imaging artefacts were discarded.

### Intracellular Cadmium Accumulation Measurements by Real-Time Fluorescence Imaging

Accumulation of intracellular Cd^2+^ in transiently transfected HEK293T cells was monitored with a FLIPR tetra microplate reader (Molecular Devices) using Calcium 5 Assay Kit (Molecular Devices) as described previously (Manatschal et al., 2019). Briefly, a baseline was recorded for 50 s, then, the indicated concentration of Cd^2+^ was added and changes in fluorescence intensity were recorded for 15 min. Transport activity was quantified as the Area Under the Curve (AUC) of the fluorescent signal induced by the Cd^2+^ influx into the cells. Average fluorescence measured in the non-transfected cells was used to subtract the non-specific background signal. Experiments were conducted in a calcium free uptake buffer (117 mM NaCl, 4.8 mM KCl, 1 mM MgCl_2_, 10 mM glucose, 10 mM HEPES, pH 7.4). The experiment was performed in duplicate the indicated independent number of times, each experimental condition was measured in 3–6 wells per plate.

### Radiolabelled Iron Uptake Assay

Accumulation of radiolabelled Fe^55^ in transiently transfected HEK293T was performed as previously described (Poirier et al., 2020). Accumulated Intracellular ^55^Fe was measured with the MicroBeta^2^ microplate counter for radiometric and luminescence detection (PerkinElmer). Determined counts per minute (cpm) where transformed into influx rates (pmol.min^−1^) as previously described (Pujol-Giménez et al., 2017). Kinetic parameters were obtained using the Michaelis-Menten equation. Average ^55^Fe measured in the non-transfected cells was used to subtract the non-specific background signal. The experiment was performed in duplicate the indicated number of independent times, each experimental condition was measured in 2–4 wells per plate.

### Surface Biotinylation

Cells expressing the proteins under study were washed once with PBS and incubated at 4°C for 1 h with 1.5 mg/ml Sulfo-NHS-SS-Biotin (ThermoFisher) in a horizontal shaker. Once the surface proteins were biotinylated, cells were washed with a quenching buffer (PBS supplemented with 1 mM MgCl_2_, 0.1 mM CaCl_2_, and 100 mM glycine) and rinsed again with PBS. Next, cells were lysed in radio immunoprecipitation assay buffer (150 mM NaCl_2_, 5 mM EDTA, 1% Triton X-100, 0.5% deoxycholate, 0.1% SDS and 50 mM Tris-HCl, pH 7.4) supplemented with fresh cOmplete™ protease inhibitor cocktail. Cell lysates were incubated overnight with streptavidin-agarose beads at 4°C. Beads were washed sequentially with solution A (50 mM NaCl, 5 mM EDTA and 50 mM Tris-HCl, pH 7.4) three times, solution B (50 mM NaCl and 50 mM Tris-HCl, pH 7.4) twice, and solution C (50 mM Tris-HCl, pH 7.4) once. Biotin bound surface proteins were released from the beads by heating at 95°C with 2X Laemmli buffer.

### Tissue Protein Isolation

Membrane fractions were isolated from kidney and lung tissues using the Mem-PER™ Plus Membrane Protein Extraction Kit (ThermoFisher 89842Y). Briefly, samples were homogenized using lysin matrix D ceramic beads (MP Biomedicals™ 116913050) with a cell permeabilization buffer, allowing the release of soluble cytosolic proteins, followed by membrane solubilization buffer to release membrane proteins. All the buffers contain protease and phosphatase inhibitors (Halt™ Protease and Phosphatase Inhibitor- 78442).

Total liver lysate was obtained from liver tissue homogenized with ceramic beads at 4°C in RIPA buffer (Sigma, R0278) containing protease inhibitors (Roche, Mannheim, Germany). Homogenates were clarified by centrifugation at 30,000 g for 10 min and protein quantified according to the kit instruction (BCA, Pierce, 23225).

### Immunoblotting

Biotin bound surface proteins were separated on 8% SDS-polyacrylamide gels, and transferred onto PVDF blotting membranes. Protein detection was achieved by incubating membranes blocked with 5% non-fat milk with the corresponding primary and secondary antibodies and detected by enhanced chemiluminescence (ECL). The following antibodies and dilutions were used: Mouse monoclonal anti-HA (1:1000), mouse monoclonal anti-Na^+^/H^+^ exchanger 1 antibody (1:1000); mouse monoclonal anti-actin (1:1000) (Santa Cruz Biotechnology); and HRP-conjugated goat anti-mouse IgG (1:3000) (Bio-Rad). To verify equal loading among samples, all the biotinylated proteins were visualized with Avidin–HRP conjugate (1:1000 dilution) (Bio-Rad). Quantification was performed using the ImageJ software (National Institutes of Health) (Schneider et al., 2012).

Total liver lysates, lung and kidney membrane fractions were separated on 10% SDS-polyacrylamide gels, and transferred to nitrocellulose membranes (Thermo Scientific 88018). Protein detection was achieved by incubating membranes, previously blocked with 10% non-fat milk and 5% BSA, with the corresponding primary and secondary antibodies and revealed by chemiluminescence using ECL solution. Following antibodies and dilutions were used: rabbit monoclonal anti-insulin receptor (InsR) (1:1000) (Cell Signaling), mouse monoclonal anti-actin (1:1000) (Santa Cruz Biotechnology), mouse monoclonal anti- GAPDH (1:1000) (Santa Cruz Biotechnology), mouse monoclonal anti-DsRed Antibody (1:1000) (Santa Cruz Biotechnology), anti-ZIP8 antibody (2 μg/ml) [gift from Dr. Knutson (Wang et al., 2012)] and e-cadherin (1:1000) (Cell Signaling).

### Mice and Diet

All animal experiments were started on 8 weeks old C57Bl/6 males and were approved by the local veterinary authorities of the Kanton Bern, Switzerland (BE65/15 and BE14/19) and conducted in accordance with the Swiss Animal Welfare Law. The *Zip8 A393* (ZIP8KI) mice were generated by PolyGene (Riedmattstrasse 9, 8153 Rümlang, Switzerland). The Ala393 (A393) which corresponds to Ala391 in human, was inserted together with a second silent mutation 8 bp downstream, resulting in the deletion of a SspI restriction without affecting the amino acid sequence. The FRT-flanked neomycin resistance cassette was inserted in intron 8 and excised by Flp recombination, upon breeding with germline Flp expressing mice. Heterozygotes breeding pairs in C57Bl/6 background were selected to produce *Zip8* wild-type (WT) and *Zip8* A393 (ZIP8KI) for the experiments.

Mice were genotyped by PCR using genomic DNA isolated from the ear using the following primers: E517.30 (5′-GCT​GCC​CTT​CAG​CTC​GAA​AC-3′) and E517.31 (5′- ACG​TCC​ATA​GCG​ATC​CTG​TG -3′). The PCR product was subsequently digested by SspI (Thermo Scientific ER0771).

Mice were housed in a temperature and humidity-controlled room with a 12 h light/dark cycle and had free access to food (standard diet (StD) # 2223, Provimi Kliba AG: iron 65 mg/kg, zinc 45 mg/kg, copper 6 mg/kg, iodine 0.6 mg/kg, manganese 12 mg/kg, selenium 0.2 mg/kg) and water. For sucrose supplementation, mice were fed in StD diet and had free access to drinking water containing 2% sucrose for 45 days.

24 h urine was collected under mineral oil from mice housed in metabolic cages after 2 days of adaptation. Terminal blood collection was performed early afternoon in 4 h starved animals under isoflurane anaesthesia in lithium heparinized tubes. Liver, kidney, lungs, and femurs were collected and frozen at −80°C until use or fixed in 4% paraformaldehyde.

### Telemetry

Arterial blood pressure (BP) was recorded by implantable telemetry (DSI, St. Paul, MN) in awake mice fed the StD diet, as described (Schuler et al., 2009). Briefly, the transducer was implanted in the carotid under isoflurane anesthesia on a heating surface set at 36°C. The body of the transmitter was positioned in an s.c. pocket on the right flank. After 1 week of recovery, transmitters were activated and offset-corrected blood pressure was recorded for 3 days. BP was measured every 1 min and the average between 8:30 (ZT3) and 15:30 (ZT10) and between 19:30 (ZT14) and 02:30 (ZT21) represented BPs in the light and dark phases, respectively. During the recording the room was maintained at 22–24°C with a 12 h light/12 h dark cycle, and mice had free access to food and water.

### Glucose and Insulin Tolerance Test

Experiments were performed in the early afternoon on mice starved for 6 h (Andrikopoulos et al., 2008). Blood glucose was measured before and after intraperitoneal injections of glucose (2 g/kg) or insulin (1 U/kg Actrapid HM, Novo Nordisk, Denmark) using a glucometer (Contour glucose monitor, Bayer Healthcare, Germany) after tail vein sampling at indicated time points. Serum insulin concentrations were determined by ELISAs (CrystalChem, Downers Grove, IL, United States).

### Plasma and Urine Biochemistry

Plasma and urinary electrolytes were determined at the core laboratory of the Bern University Hospital, Bern, Switzerland. HDL-C and LDL-C/VLDL-C were measured in plasma with a colorimetric method using BioVision’s HDL-C and LDL-C/VLDL-C Quantification Kit (#K613-100).

### Metal Ion Analysis

Trace elements in plasma and tissues were measured by inductively coupled plasma mass spectrometry (ICP-MS) (7700 Series; Agilent, Palo Alto) as described elsewhere (Jafari et al., 2018; Daruich et al., 2019). Prior to analysis, samples were diluted with a 1% nitric acid solution containing 10 ng/ml rhodium and 10 ng/ml indium as internal standards. Each analytical batch of study samples was processed with laboratory controls, including method blanks and standard reference materials, to continuously monitor method performance.

### MicroCT Scan

Bones were fixed in 4% paraformaldehyde in phosphate-buffered saline for 24 h and subsequently transferred to 70% ethanol for MicroCT analysis (Scanco Medical AG, Brüttisellen, Switzerland) with the following parameters: the X-Ray source (E) was set at 70 kVp, with 177 μA at High Resolution (1000 Projections/180°), which showed an Image Matrix of 2048 × 2048 pixels; the diameter of the sample holder was 12 mm, which allowed an increment (Resolution) of 6 μm (=Voxelsize); integration time was set on 300 ms.

The evaluation of the reconstructed 2D Images was made with a 3D morphometry of VOI (Volume of Interest,“Bone Trabecular Morphometry”, script from Scanco Medical AG), Gauss Sigma at 0.8 and Gauss support at 1, a lower threshold at 0 and upper threshold at 200 of a 1/1000 scale.

### Real-Time PCR

Total RNA was extracted from kidney or liver tissues using Trizol. cDNA was synthesized from 500 ng total RNA by reverse transcriptase (PrimeScript RT Master Mix; Takara) with oligo dT primers. Real-time PCR was performed with the primers and probes from Roche (*slc30a8*: F: 5′-CTG​ATG​CGG​CTC​ATC​TCT​TA-3′, R: 5′-CCT​CGA​TGA​CAA​CCA​CAA​AG-3′, probe: 53. *Beta-actin*: 4352341E Thermofischer) on a 7500 Fast Real-Time PCR System. Ct values for duplicate technical replicates were averaged, and the amount of mRNA relative to β-actin was calculated using the ΔCt method.

### Statistical Analysis

Normality of the distribution of the different data sets was evaluated using Kolmogorov-Smirnov and Shapiro-Wilk test. Comparisons were established using unpaired *t*-test or Mann-Whitney, depending on the distribution. Analysis was also established with One-way Anova and comparisons were established Tukey or Fisher’s LSD. Statistical analysis was performed using the IBM SPSS statistics 20 software and Graph Pad Prism 8.

## Results

### The Human ZIP8 A391T Variant has Reduced Metal Ion Transport Capacity and is Less Expressed *In Vitro* Compared to WT

**FIGURE 1:**
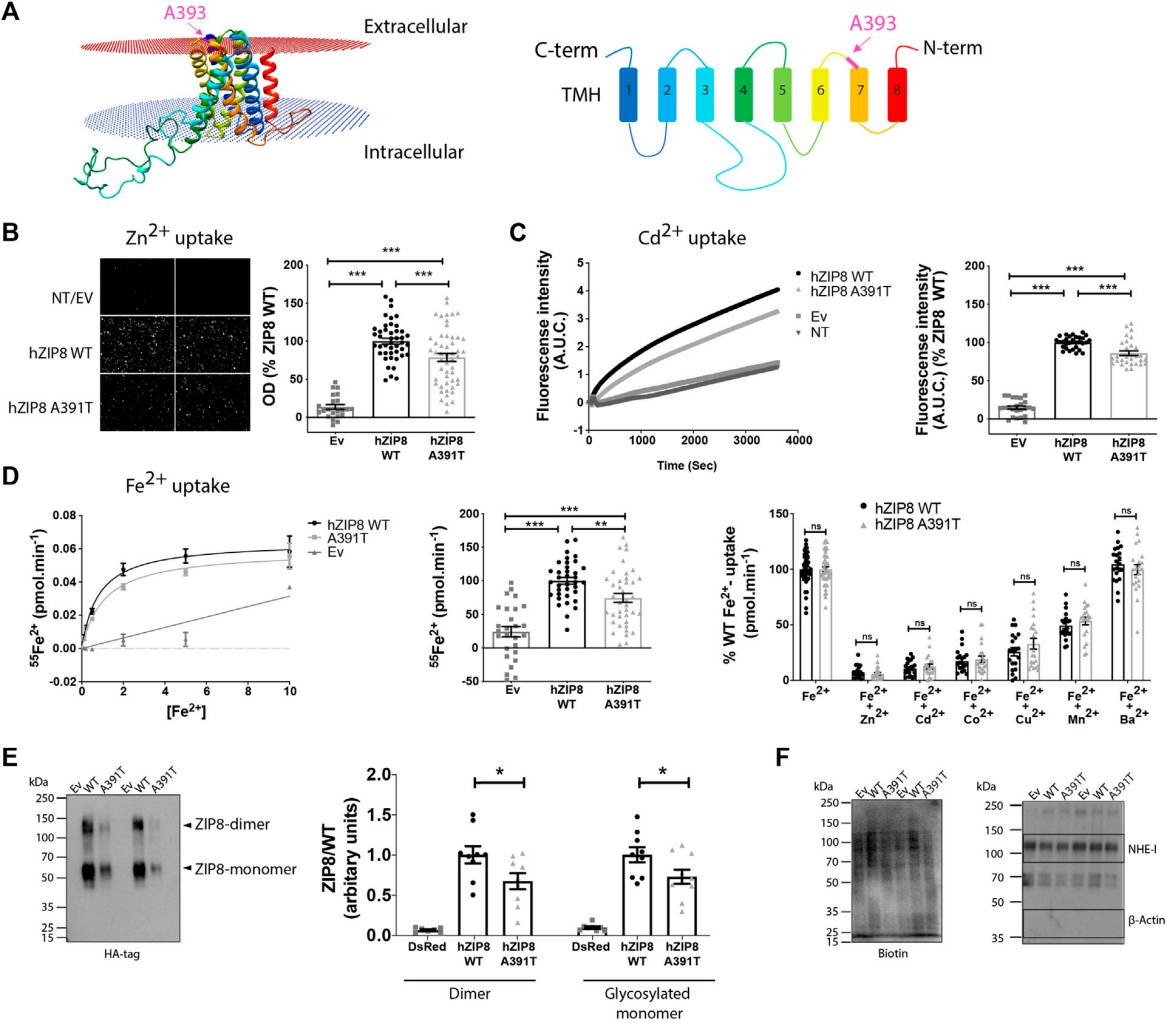
Murine ZIP8 homology model, metal ion transport and plasma membrane expression of human ZIP8 WT and A391T mutant in transiently transfected HEK293T cells. **(A)** Left panel: 3D structure of mouse ZIP8 based on the bbZIP X-ray structure (Zhang et al., 2017). Right panel: Diagram indicating the disposition of C and N-terms and the colouring and numbering of the 8 Transmembrane Helix (TMH). **(B)** Left panel: Representative fluorescence microscopy images of intracellular Zn^2+^ (10 μM) accumulation. Right panel: Normalized values from six independent experiments (*n* = 22–51) are represented individually. **(C)** Left panel: Representative experiment showing the change on fluorescence intensity as result of intracellular Cd^2+^ (10 μM) accumulation. Right panel: Normalized values from four independent experiments (*n* = 2–30) are represented individually. **(D)** Left panel: Representative experiment showing ^55^Fe^2+^ transport kinetics [0.1–10 μM]. Middle panel: Normalized values of iron transport (10 μM) obtained from five independent experiments (*n* = 27–40) are represented individually. Right panel: Iron transport (1 μM) in the presence of an excess of different divalent metals (Zn^2+^, Cd^2+^, Co^2+^, Cu^2+^, Mn^2+^ and Ba^2+^) (10 μM). Normalized values from independent experiments (*n* = 2) are represented individually. **(E)** Representative blot showing the plasma membrane surface protein expression determined using an anti-HA monoclonal antibody. Normalized results obtained from three independent experiments, performed in duplicate. **(F)** Left panel: Biotin content of each sample as loading control. Right panel: Plasma membranes expression of Na^+^/H^+^ exchanger (NHE-1) and lack of β-actin expression are shown as control of membrane surface samples purity. All the data are mean ± SEM of the indicated number of biological replicates. Statistical differences between groups were assessed using either *t*-test or Mann-Whitney U according to the sample distribution. Significance was set at *p* < 0.05; ^∗∗^ *p* < 0.01; ^∗∗∗^ *p* < 0.001. NT, non-transfected; EV, empty vector.

SNP rs13107325 located in exon 8 of SLC39A8 is characterized by the major allele having a cytosine (C) and the minor allele having a thymidine (T) at the first position of the codon for amino acid 391. Ala391 in human corresponds to Ala393 (A393) in mouse. Thus, in mice, the protein ZIP8 can either be the small size and hydrophobic Ala393 (encoded by the C allele) or the medium size and polar Thr393 residue containing variant (encoded by the T allele). To understand the effect of this mutation on ZIP8 function, we generated a homology-based 3D model of the mouse ZIP8 protein (Figure 1A). In our model, Ala393 is located in an extracellular loop between transmembrane helix 6 and 7, away from the putative metal binding site located within the intramembrane hydrophilic cavity. Due to its remote location, we hypothesize that this mutation does not change the transport properties of ZIP8.

To test this hypothesis, we evaluated the effect of the ZIP8 variant A391T on zinc (Zn^2+^), cadmium (Cd^2+^) and iron (Fe^2+^) transport *in vitro*, using HEK293T cells overexpressing either human ZIP8 WT or A391T (Figures 1B–D; Supplementary Figure S1). For all three divalent metal ions, there was a significant decrease in uptake by ZIP8 A391T variant using both native (Figures 1B–D) and HA-tagged constructs (Supplementary Figures S1A–C). In order to rule out a possible effect of the mutation on the main transport properties of ZIP8, we next examined the transport kinetics and substrate selectivity of both WT and A391T ZIP8. In line with our hypothesis, the affinity for Fe^2+^ (Km _WT=_ 0.82 ± 0.01 μM vs. Km _A391T_ = 1.01 ± 0.2 μM) and selectivity for substrates remained unaltered in the mutant ZIP8 (Zn^2+^ > Cd^2+^ > Co^2+^ > Cu^2+^ > Mn^2+^) (Figure 1D).

Since the main transport properties of A391T ZIP8 remained unchanged, we next assessed the amount of functional ZIP8 protein, which is present as both monomers and dimers. We quantified HA-tagged WT and A391T mutant ZIP8 on the plasma membrane in transiently transfected HEK293T cells. The mutant variant showed a significant decrease in surface membrane expression for both monomer and dimer (≈30%) (Figure 1E) while all the samples had similar loading amounts (Figure 1F) and transfection efficiencies (Supplementary Figure S1D), a result which corresponds to the observed decrease in uptake of divalent metal ions in the range of 15%–34% (Figures 1B–D).

Overall, our *in vitro* studies indicate that the A391T mutation causes reduced surface expression of the protein, which subsequently reduces its transport capacity to the same extent, without altering the general transport properties.

### Zip8 A393T (ZIP8KI) Mice Exhibit Lower Protein Expression and Thus Abnormal Metal Ion Homeostasis

**FIGURE 2:**
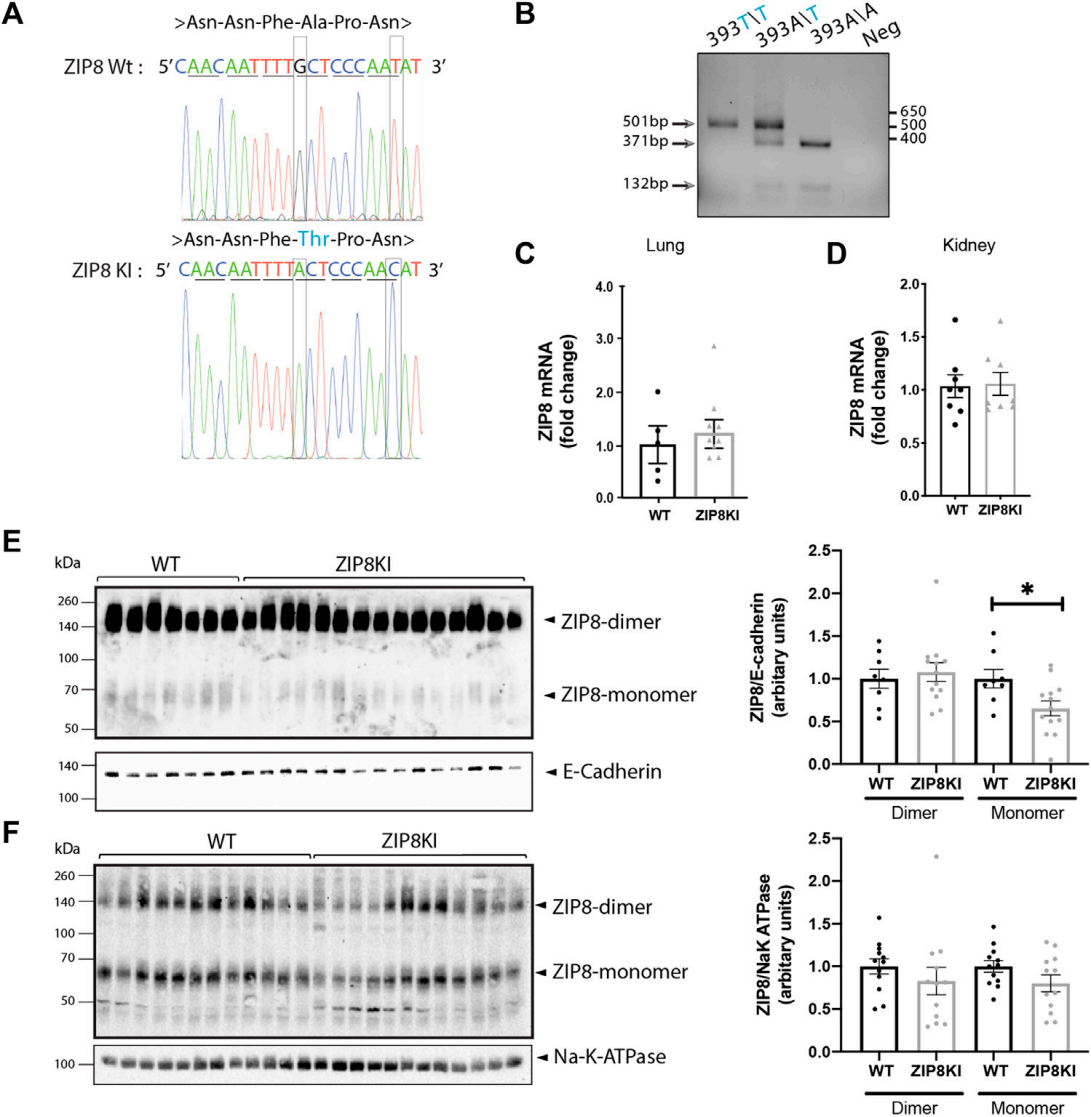
ZIP8KI mice express less ZIP8 protein. **(A)** Sequencing analysis for A393T mutation in the Slc38A9 mice. The 1177G>A mutation in exon 8 results in an exchange of alanine (Ala) to threonine (Thr, blue). For detection of the mutated allele in the genome the SspI restriction site is deleted by a silent exchange of 1185T>C. **(B)** Genotyping for the detection of the A393T mutation in the Slc39A8 locus. DNA extracted from ear biopsies of mice. The expected PCR signal of 503 bp is cut into two fragments of 371 and 132 bp in the wild type, and the mutant allele remain uncut fragment of 503 bp. The homozygote mouse shows the expected cleavage products, demonstrating introduction of the mutation into the genome. **(C)** Relative mRNA expression of *slc39a8* in lung and **(D)** kidney of WT and ZIP8KI male mice normalized to actin under StD diet (*n* = 8). **(E)** Western blot analysis and quantification of ZIP8 protein expression in lung and **(F)** kidney membrane extracts in WT and ZIP8KI (*n* = 5–15) Means ± SEM of animals in each experimental condition. ^∗^ *p* < 0.05 comparisons made between WT and ZIP8KI mice.

The A393 variant in mice corresponding to the human genetic variant in ZIP8 (rs13107325; A391T) was produced by homologous recombination. The success of the model was confirmed by DNA sequencing of WT and ZIP8KI mice (Figure 2A). Heterozygote mice were interbred to obtain WT and homozygote ZIP8KI mice which were born according to Mendelian ratios. ZIP8KI variant was detected by digestion of a targeted PCR fragment with the enzyme Ssp1 (Figure 2B).

We next evaluated the expression of the ZIP8 transcript and protein in lungs, where ZIP8 shows the highest levels of expression, as well as in the kidney, as ZIP8 plays an important role in metal reabsorption from the glomerular filtrate, which could affect the overall metal homeostasis. The mRNA expression levels of ZIP8 in lung (Figure 2C) and kidney (Figure 2D) homogenates remained unchanged. In lung expressing very high amounts of the protein, there was a statistically significant (−35%) reduction of glycosylated ZIP8 monomer in ZIP8KI compared to WT mice (Figure 2E) and a similar tendency was observed in kidney (Figure 2F).

**FIGURE 3:**
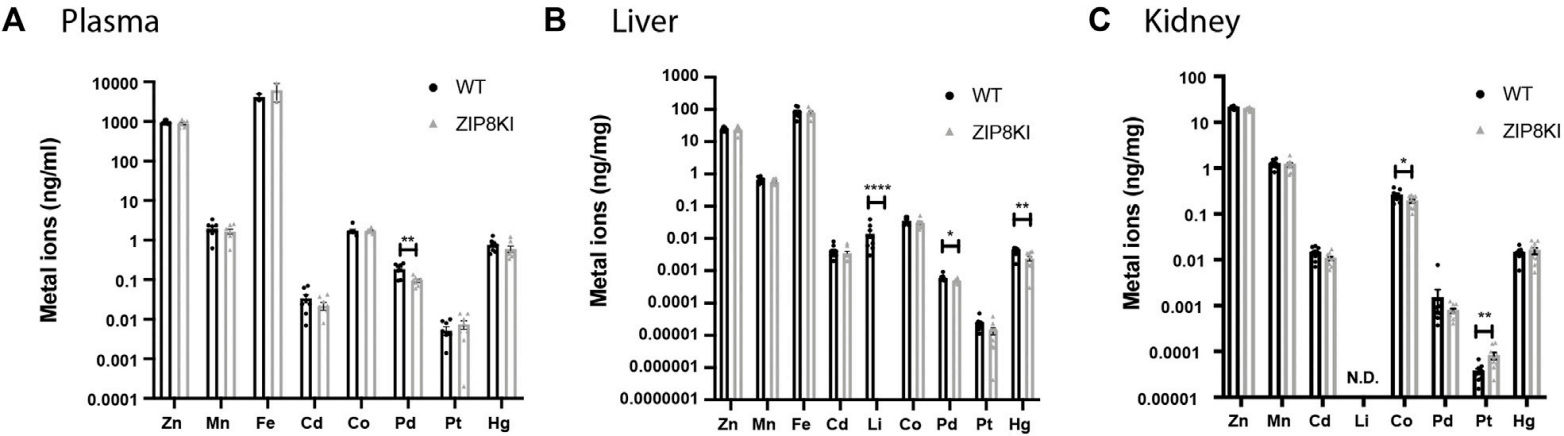
ZIP8KI mice harbour abnormal metal ion composition. Metal ion composition was analysed by ICP-MS in **(A)** plasma, **(B)** liver and **(C)** kidney extracts from WT and ZIP8KI mice (*n* = 8) Means ± SEM of animals in each experimental condition. ^∗^ *p* < 0.05, ^∗∗^ *p* < 0.01, ^∗∗∗∗^ *p* < 0.0001 comparisons made between WT and ZIP8KI mice.

Given the broad substrate selectivity of ZIP8, we quantified a variety of metal ions in plasma, liver and kidney of WT and ZIPKI mice fed with StD (Figure 3; Supplementary Table S1). ZIP8KI mice had lower levels of palladium (Pd) in plasma (−49%, *p* = 0.002) (Figure 3A) and liver (−8%, *p* = 0.03) compared to WT (Figure 3B). In addition, in liver, lithium (Li) became undetectable in ZIP8KI and mercury (Hg) (−40%, *p* = 0.006) was significantly reduced compared do WT (Figure 3B). In kidney homogenates, cobalt (Co) was significantly reduced in ZIP8KI (−25%, *p* = 0.05), whereas platinum (Pt) increased more than twice (200%, *p* = 0.01) (Figure 3C). All the other metal ions including manganese (Mn), zinc (Zn) and iron (Fe) remained unchanged (Figure 3; Supplementary Table S1).

### ZIP8KI Mice Have Lower Blood Pressure and Normal Plasma Lipid Composition

To address the impact of A393T in mouse physiology, we first analyzed several metabolic parameters in animals fed with StD. The polymorphism had no significant effect on body weight, organ weight, food consumption, water intake and urinary volume (Supplementary Table S2). Next, we quantified essential biochemical parameters in plasma and 24 h urine collected from mice kept in metabolic cages (Table 1). Higher phosphate levels were detected in the plasma of ZIP8KI mice, while they excreted more glucose in urine when compared to WT. Despite the phosphate changes, the bone structure remained unchanged at the time point investigated (Supplementary Figure S2).

**TABLE 1:** Hyperphosphatemia and glucosuria in ZIP8KI mice.

Plasma	WT	ZIP8KI	*p* value
Na^+^ (mmol/L)	142 ± 2.12 (7)	140 ± 1.33 (8)	0.48
K^+^ (mmol/L)	4.49 ± 0.21 (7)	4.26 ± 0.09 (8)	0.31
Cl^−^ (mmol/L)	91.21 ± 1.03 (7)	97.50 ± 0.63 (8)	0.0008***
Ca^2+^ (mmol/L)	2.28 ± 0.04 (7)	2.26 ± 0.05 (8)	0.8
P (mmol/L)	1.95 ± 0.09 (7)	2.26 ± 0.07 (8)	0.02*
Mg (mmol/L)	1.05 ± 0.07 (7)	0.95 ± 0.05 (8)	0.27
CaxP (mm^2^)	4.44 ± 0.21 (7)	5.09 ± 0.16 (8)	0.03*
Hematocrit (%)	55.9 ± 0.68 (10)	55.0 ± 1.77 (8)	0.61
Glucose (mmol/L)	4.96 ± 0.80 (8)	5.94 ± 1.00 (7)	0.45
HDL (μg/ml)	56.68 ± 7.99 (8)	46.79 ± 9.76 (8)	0.45
LDL (μg/ml)	51.39 ± 7.86 (8)	51.39 ± 7.86 (8)	0.24

Urine	WT	ZIP8KI	*p* value
Urinary pH	5.7 ± 0.05 (8)	6.0 ± 0.05 (8)	0.41
Na^+^/Cr (μmol/μmol)	45.9 ± 5.6 (10)	55.2 ± 2.7 (14)	0.12
K^+^/Cr (μmol/μmol)	66.1 ± 4.3 (10)	69.9 ± 3.5 (14)	0.50
Cl^−^/Cr (μmol/μmol)	56.2 ± 5.6 (10)	64.9 ± 2.6 (14)	0.12
Ca^2+^/Cr (μmol/μmol)	1.3 ± 0.37 (10)	0.56 ± 0.068 (14)	0.04*
P/Cr (μmol/μmol)	19.1 ± 1.8 (10)	19.8 ± 1.4 (14)	0.77
Mg/Cr (μmol/μmol)	5.4 ± 0.4 (5)	6.1 ± 0.49 (4)	0.32
Glucose/Cr (μmol/μmol)	1.3 ± 0.3 (10)	1.7 ± 0.2 (14)	0.005**
Urea/Cr (mmol/μmol)	0.49 ± 0.02 (5)	0.47 ± 0.02 (4)	0.51

Biochemical parameters measured in plasma and 24 h urine of male WT and ZIP8KI mice fed a standard diet. Data shown are means ± SEM (n). *p < 0.05, **p < 0.01, ***p < 0.005 comparisons made between WT and ZIP8KI mice.

**FIGURE 4:**
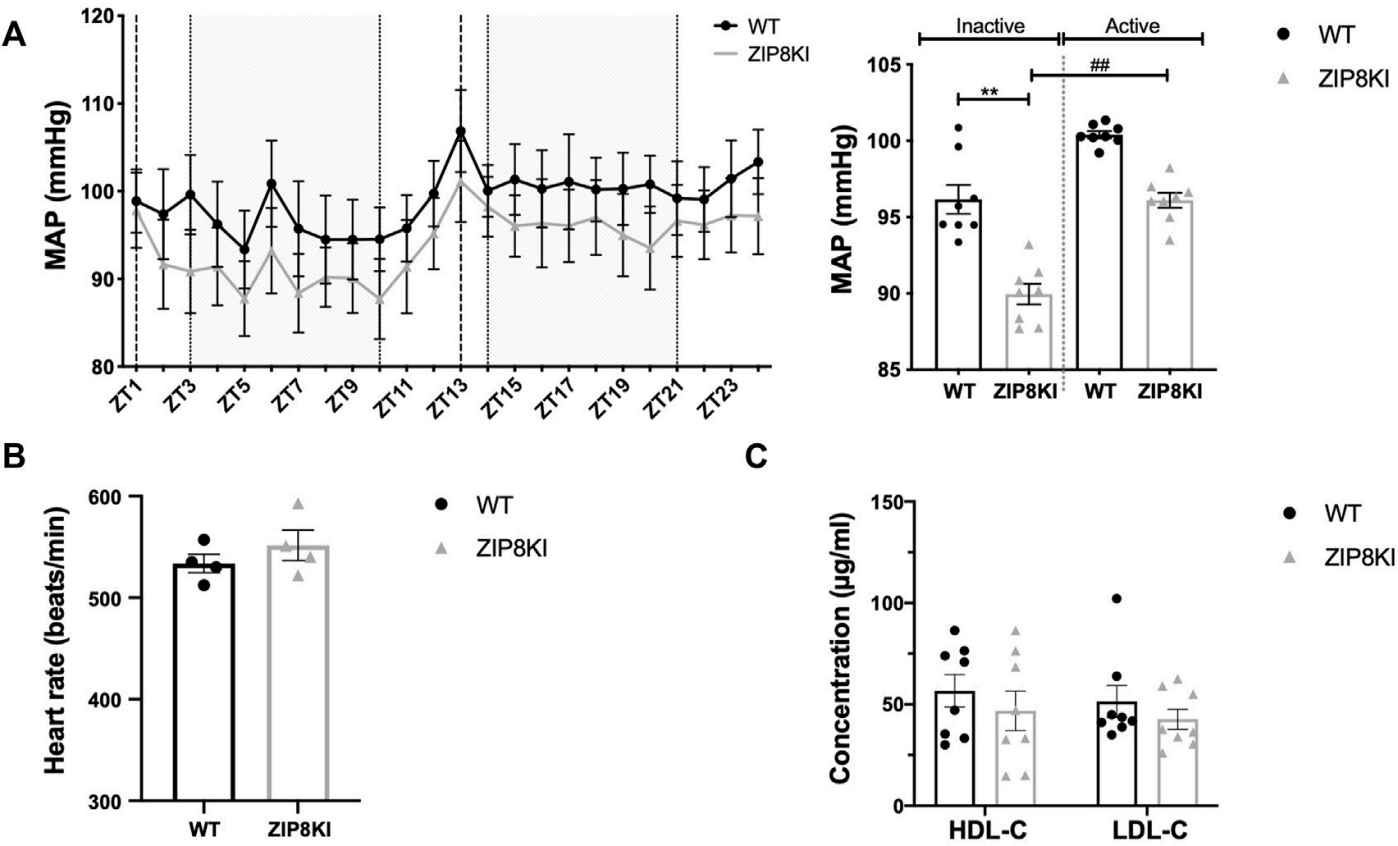
ZIP8KI have lower blood pressure and normal plasma lipid composition. Blood pressure was measured by telemetry for 24 h in WT and ZIP8KI mice fed with StD diet (*n* = 4). **(A)** Mean arterial pressure (MAP) over 24 h and MAP in inactive (ZT3-ZT10) and active period (ZT14-ZT21) **(B)** heart rate. Plasma lipid composition **(C)** HDL-C and LDL-C in WT and ZIP8KI mice fed with StD diet. Means ± SEM of animals in each experimental condition, (*n* = 7–8). ^∗∗^ *p* < 0.01comparisons made between WT and ZIP8KI mice. ^##^ *p* < 0.01 comparisons made between mice with the same genotype.

**FIGURE 5:**
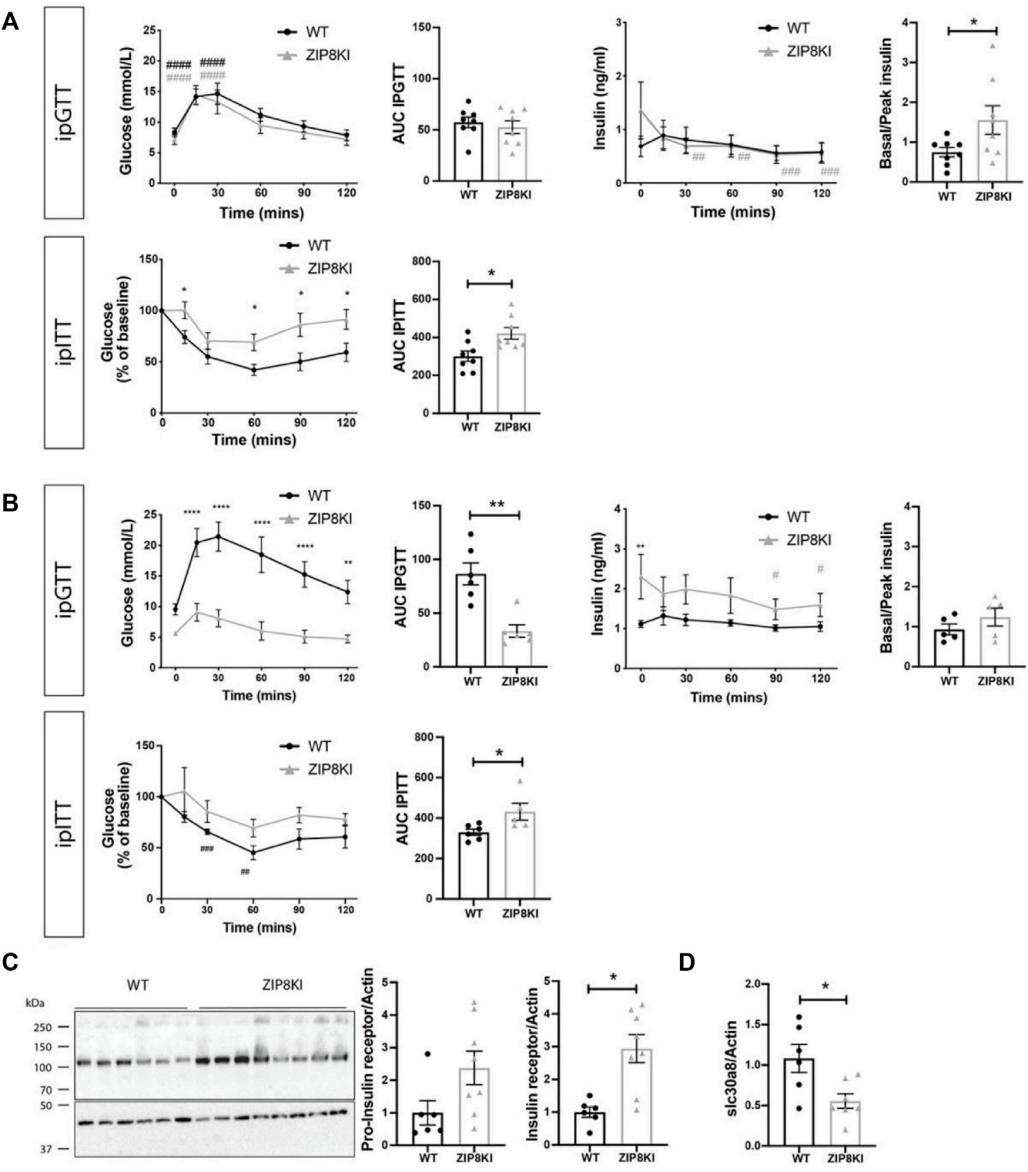
ZIP8KI are more insulin resistant in StD diet and protected against increased blood glucose levels. ZIP8KI express higher hepatic Insulin Receptor and higher transcript levels of slc30a8 after treatment with sucrose. **(A)** Intraperitoneal glucose tolerance test (ipGTT) in mice fed with StD diet (*n* = 8). Glucose (mmol/L) and insulin (ng/ml) levels were measured in fasting state (6 h) and 15, 30, 60, 90 and 120 min after administering glucose (2 g glucose/kg). The area under curve for glucose measurements waand basal to peak insulin ratio (after 15 min) were calculated. In intraperitoneal insulin tolerance test (ipITT) in mice fed with StD diet (*n* = 8) glucose (mmol/L) levels were measured in fasting state (6 h) or 15, 30, 60, 90 and 120 min after administering insulin solution (1U/kg). The area under curve for glucose measurements was calculated. **(B)** Intraperitoneal glucose tolerance test (ipGTT) (*n* = 6) and intraperitoneal insulin tolerance test (ipITT) (*n* = 6) in mice fed for 45 days with 2% sucrose in water (Figure 4A) **(C)** Western blot and its quantification of pro-insulin and insulin receptor expression in liver of WT and ZIP8KI mice fed for 45 days with 2% sucrose in water (*n* = 6–8). Relative mRNA levels of **(D)** *slc30a8* in liver of WT and ZIP8KI male mice (*n* = 6–8) normalized to actin fed for 45 days with 2% sucrose in water (*n* = 8). ^∗^ *p* < 0.05, ^∗∗^ *p* < 0.01 comparisons made between WT and ZIP8KI mice. #*p* < 0.05, ##*p* < 0.01, ###*p* < 0.005, ####*p* < 0.001 comparisons made between *t* = 0 and other time points for the same genotyping.

Given the association of this polymorphism with lower BP and reduced HDL-C in GWAS (Costas, 2018; Parisinos et al., 2020), we measured BP by implantable telemetry and analyzed the lipid profile in ZIP8KI mice (Figure 4). ZIP8KI mice had significantly lower mean BP than the WT mice, both during the active (*p* = 0.005) and inactive phase (*p* = 0.05) (Figure 4A). The heart rate remained unchanged (Figure 5B). ZIP8KI and WT mice had similar HDL-C and LDL-C levels (Figure 4C).

### ZIP8KI Mice are Protected From Increased Glucose Levels

Due to observed increased glycosuria in ZIP8KI mice and since other members of ZIP and ZnT families have been reported to be implicated in the regulation of blood glucose (Bellomo et al., 2011), we evaluated the impact of this ZIP8 polymorphism on glucose homeostasis. We performed intraperitoneal glucose tolerance test (ipGTT) and insulin tolerance (ipITT) test in WT and ZIP8KI mice first fed with StD and then challenged with sucrose-containing diet.

Under StD, ZIP8KI mice had similar glucose levels compared to WT after starving for 6 h (t = 0), but they tended to have higher insulin levels (Figure 5A). Following glucose injection (t = 30 min), blood glucose level reached 15 mmol/L and returned to baseline after 2 h in both WT and ZIP8KI mice. The area under the curve (AUC) calculated for ipGTT showed no significant difference between WT and ZIP8KI mice. Insulin was measured in parallel to glucose during ipGTT. The ratio basal/peak insulin during the ipGTT calculated after 15 min was increased (*p* = 0.02) in ZIP8KI mice (Figure 5A upper panel). Following insulin injection (ipITT), glucose levels dropped in WT mice but remained higher in ZIP8KI mice (Figure 5A lower panel). This was reflected by a significantly increased AUC, suggesting that ZIP8KI mice have insulin resistance (Figure 5A lower panel).

We repeated ipGTT and ipITT tests in mice challenged by sucrose consumption in drinking water for 45 days (Figure 5B). After 6 h starvation (t = 0), WT mice had higher glucose levels compared to StD diet (from 7 to 10 mmol/L) and during ipGTT the blood glucose increased up to 21 mmol/L (Figure 5B upper panel). In contrary, baseline glucose (t = 0) for ZIP8KI mice after sucrose consumption remained unchanged when compared to ZIP8KI mice fed with StD and, during ipGTT, and blood glucose increased only up to 9 mmol/L (Figure 5B upper panel). The AUC for glucose was more than twice lower in ZIP8KI mice compared to WT mice (Figure 5B upper panel). Insulin measurements during ipGTT revealed that ZIP8KI had a greater compensatory ability to control glucose levels already after starvation (t = 0) due to increased insulin secretion, which was statistically significant at this time point (Figure 5B lower panel) and remained higher during the test. Following insulin injection in ipITT, plasma glucose dropped in ZIP8 WT and the effect was less pronounced in ZIP8KI mice, leading to increased AUC for glucose. These results indicate that ZIP8KI mice remain insulin resistant following addition of sucrose in drinking water (Figure 5B).

To pinpoint possible underlying mechanism accounting for these changes, we conducted studies on the liver as a key organ involved in regulating metal ion homeostasis. We quantified the amount of pro-insulin receptor and insulin receptor (Figure 5C). We found a 2-3-fold higher expression of both forms in ZIP8KI mice after sucrose treatment compared to WT. We also analyzed the hepatic transcription levels of *slc30a8*, a zinc transporter related to insulin secretion in human (Kleiner et al., 2018). The later was downregulated by 50% in ZIP8KI mice (Figure 5D).

## Discussion

The ZIP8 variant rs13107325 (A391T) is considered to be one of the most pleiotropic SNPs in the human genome. GWAS studies suggested an association of this SNP with reduced BP, hyperlipidaemia, and increased body mass index. We performed an *in vitro* characterization of the functional changes induced by the A391T mutation on the ZIP8 transport activity and created the ZIP8KI mouse model to analyse the changes in physiology and metabolism.

The precise role of this polymorphism in metal ion transport and protein expression is controversial. An earlier *in vitro* study indicated that Fe^2+^ or Zn^2+^ transport in cells transfected with A391T ZIP8 was not impaired when compared to WT, but Mn^2+^ transport was significantly reduced (Haller et al., 2018). In a second study, it was shown that A391T ZIP8 has a reduced ability to transport Cd^2+^ without changing the protein expression level (Zhang et al., 2016). A third study suggested a lower expression level of A391T ZIP8 (Melia et al., 2019), but the metal transport activity was not measured. Thus, we designed *in vitro* experiments to clarify these discrepancies. Using HEK293T cells, we show that the A391T ZIP8 exhibits reduced plasma membrane protein expression, which is accompanied by a corresponding decrease in its capacity to transport divalent metals. However, there were no significant changes between mutant and WT in terms of transport kinetics and substrate selectivity. In line with this, the generated ZIP8 3D homology model revealed that Ala391 is part of an extracellular loop, far away from the predicted substrate binding site. This further supports the concept that the mutation is unlikely to change metal-binding ability. *In vivo*, we also observed a lower plasma membrane expression in the lungs and a similar tendency in the kidneys while, at the mRNA level, we did not observe discernable changes in the kidneys of ZIP8KI mice compared to WT.

A previous study showed that, under iron loading conditions, total ZIP8 protein levels increased while ZIP8 mRNA levels remained unaltered, suggesting a post-transcriptional regulatory mechanism (Wang et al., 2012). It is tempting to predict that a similar post-translational regulatory mechanism exists for the ZIP8 mutant that alters expression at the protein level but not at the mRNA level. Whether this change in expression would be the result of altered protein folding, trafficking, insertion in the membrane or disruption of a regulatory site, remains to be clarified.

GWAS suggested that A391T ZIP8 is associated with lower levels of circulating Mn (Ng et al., 2015; Mealer et al., 2020). In previous studies, it was also described that ZIP8KI mice have lower Mn levels without changes in Zn and Fe levels in blood, kidney and liver (Nakata et al., 2020; Sunuwar et al., 2020). The concentrations for these three metal ions remained unchanged in plasma, kidney and liver in our study. This inconsistency between the two *in vivo* studies could be explained by the significant differences in diet composition. In our study we used a diet containing much lower amounts of metal ions (manganese 12 mg/kg, zinc 45 mg/kg, iron 65 mg/kg) compared to the other study (manganese 100 mg/kg, zinc 70 mg/kg, iron 200 mg/kg) (Sunuwar et al., 2020).

Interestingly, under our experimental conditions, we observed changes in the tissue levels of other metal ions such as mercury in the liver and cobalt and platinum in the kidneys. Of these three metal ions, only cobalt has so far been identified as a substrate of ZIP8 (Wang et al., 2012; Nebert et al., 2012; He et al., 2006), as also shown by our metal competition experiments **(** Figure 1D **)**. Previous studies suggested that the functional role of ZIP8 is not limited to the transport of Mn^2+^, Fe^2+^ or Zn^2+^ (Wang et al., 2012). Our results support the hypothesis that ZIP8 can significantly contribute to the homeostasis of other divalent metal ions.

We investigated in detail two of the traits that have been reported in GWAS for ZIP8 rs13107325, reduced arterial BP and altered plasma lipid composition (Costas, 2018). In our study, BP measured by implantable telemetry in ZIP8KI mice was significantly reduced, a result in agreement with the reported GWAS (Costas, 2018). A possible factor that might contribute to the reduction of BP could be palladium. Mononuclear palladium complexes can modify the proteolytic activity of enzymes of the renin-angiotensin system (Illán-Cabeza et al., 2013), and plasma palladium concentration was decreased in ZIP8KI mice.

Unlike in humans, the ZIP8KI mice kept under standard diet had HDL-C and LDL-C levels that were like those of WT mice. Indeed, other animal models like the *Slc39a8* KO and liver-specific–knockout (ZIP8-LSKO) mice also exhibited no differences in body weight and HDL-C, when compared to their WT littermates (Kim et al., 2014; Lin et al., 2017). This difference between the human study and the mouse models could be explained by a difference in lipid metabolism. The experimental conditions themselves could also be responsible for these different results, since the animals used in our experiments were of the same age, corresponding to young adults, and were fed a diet that included a fixed amount of cholesterol and fat, in addition to a fixed amount of zinc and manganese.

The ZIP8 SNP also has a remarkable impact on glucose homeostasis that was not revealed in GWAS. In our experiments, ZIP8KI mice were protected from elevated blood glucose levels and secreted more insulin in response to a hypercaloric diet. It is known that trace metals such as zinc, cadmium and mercury, when fed at physiologically normal levels, play a key role in glucose homeostasis, as they act as cofactors in glucose metabolic pathways, pancreatic β-cell function and insulin signalling cascades (Chen et al., 2009; Khan and Awan, 2014). We observed that glucose-lowering effect of exogenous insulin in ZIP8KI mice was reduced during ipITT, suggesting that ZIP8KI mice are less insulin sensitive. This finding is of great clinical significance. The change in insulin resistance could be linked to function of pancreatic islets. The correlation between other ZIP family members and glucose metabolism has already been described (Bellomo et al., 2011). In mouse pancreatic islets, elevated glucose concentrations have been shown to induce ZIP8 expression (Bellomo et al., 2011). In human, analysis of islets from type 2 diabetic donors displayed downregulation of ZIP6, ZIP7, ZIP8 and ZIP14 mRNA (Marselli et al., 2010; Dominguez et al., 2011). The role of ZIP14 in glucose homeostasis (Jenkitkasemwong et al., 2012) has been highlighted in ZIP14 knockout (KO) mice which display hyperinsulinemia and impaired insulin secretion when fed a high glucose diet (Beker Aydemir et al., 2012). In addition, a possible effect of platinum on insulin-resistance cannot be ruled out either, since the platinum level in the ZIP8KI mice increased more than twice. Consistent with this, an earlier study showed that children who survived cancer treatment with platinum-based therapy were more prone to developing insulin resistance (Baker et al., 2013).

To better understand the effect of ZIP8 on glucose metabolism, we investigated the expression of the insulin receptor β and the diabetes-susceptible hepatic zinc transporter ZnT8 (*slc30a8*) in the liver of ZIP8KI and WT mice. The insulin receptor β is predominantly expressed in tissues that are associated with insulin-dependent metabolic effects such as liver, muscle and adipose tissue, and in the diabetic condition, there is fewer insulin receptors in the liver, because their breakdown is increased (Meakin et al., 2018). As expected, we found that, under sucrose treatment, the insulin receptor expression was higher in ZIP8KI mice which had lower blood glucose. It is known that a loss of function mutation in *Slc30a8* has a protective effect against diabetes, by enhancing insulin secretion (Dwivedi et al., 2019). In our case, ZIP8KI mice showed lower *Slc30a8* expression levels, suggesting that the ZIP8 SNP might reduce ZnT8 expression as a protective mechanism against diabetes in animals with sucrose in the drinking water. A potential limitation of our study is that all experiments were performed with male mice, and it remains to be determined whether there are potential sex differences in mice or humans.

The precise clinical implications of the allelic variant ZIP8 A391T in human have not yet been assessed, despite the reported association with specific traits such as lower blood pressure in the GWAS (Costas, 2018). Searching routinely for the ZIP8 A391T variant might be relevant in patients with lower blood pressure, especially with orthostatic hypotension. Similar efforts could be useful in diabetes patients with insulin resistance, especially those with glucose intolerance.

In conclusion, our study reveals that the intrinsic transport properties of ZIP8 A391T are unchanged, while the plasma membrane expression of this variant is reduced, both *in vivo* and *in vitro*. ZIP8KI mice that carry the A391T polymorphism under standard feeding conditions exhibit strikingly altered tissue metal ion compositions and reduced arterial blood pressure. Furthermore, ZIP8KI mice exhibited insulin resistance and showed enhanced protection against increased blood glucose levels.

## Data Availability

The datasets presented in this study can be found in online repositories. The names of the repository/repositories and accession number(s) can be found below: https://figshare.com/s/628179df229ceb472160; https://figshare.com/s/d662a262dda51f034b2a.

## Abbreviations

GWAS, Genome-Wide Association Studies; SNP, Single-Nucleotide Polymorphism; ipGTT, Intraperitoneal Glucose Tolerance Test; ipITT, Intraperitoneal Insulin Tolerance Test; BP, Blood Pressure; HDL-C, High-density lipoprotein cholesterol; LDL-C, Low-density lipoprotein cholesterol; ICP-MS, Inductively Coupled Plasma — Mass Spectrometry; StD: Standard Diet; AUC: Area Under Curve.
