# Supplementary material for: The Allelic Variant A391T of Metal Ion Transporter ZIP8 (SLC39A8) Leads to Hypotension and Enhanced Insulin Resistance
Source: Front Physiol. 2022 Jun 15;13:912277. doi: 10.3389/fphys.2022.912277 (PMC9240775; doi:10.3389/fphys.2022.912277)
Supplement: Supplementary file 1 [file Table1.docx]

## **Supplementary Table 1**

**Supplementary Table 1** Metal ion composition in plasma, liver and kidney in WT and ZIP8KI mice. Data shown are means±SEM (n).

|  | Plasma | |  |
| --- | --- | --- | --- |
| Metal ions (ng/ml) | WT | ZIP8KI | P value |
| Be | 0.0023±0.0011 (8) | 0.0015±0.0004 (8) | 0.51 |
| Al | N.D. | N.D. | N.D. |
| **V** | 0.98±0.05 (8) | 0.95±0.24 (8) | 0.90 |
| **Cr** | 0.42±0.24 (8) | 0.24±0.09 (8) | 0.49 |
| **Ni** | N.D. | N.D. | N.D. |
| **Cu** | 466.8±18.9 (8) | 504.8±31.8 (8) | 0.32 |
| **As** | 0.05±0.03 (8) | 0.03±0.01 (8) | 0.56 |
| **Se** | 371.1±9.9 (8) | 381.8±11.1. (8) | 0.48 |
| **Mo** | 4.1±0.4 (8) | 3.8±0.8 (8) | 0.76 |
| **Ag** | 0.0074±0.0034 (8) | 0.0038±0.0021 (8) | 0.38 |
| **Sn** | 0.0098±0.0098 (8) | 0.0480±0.0480 (8) | 0.45 |
| **Sb** | 0.0054±0.0038 (8) | 0.0081±0.0044 (8) | 0.64 |
| **Tl** | 0.0032±0.0009 (8) | 0.0040±0.0016 (8) | 0.64 |
| **Pb** | 0.0116±0.0058 (8) | 0.0313±0.0194 (8) | 0.35 |
| **Bi** | 0.21±0.05 (8) | 0.23±0.05 (8) | 0.83 |
|  | Liver | |  |
| Metal ions (ng/mg) | **WT** | ZIP8KI | P value |
| Be | N.D. | N.D. | N.D. |
| Al | 0.69±0.11 (9) | 0.52±0.18 (10) | 0.43 |
| V | 0.0039±0.0005 (9) | 0.0032±0.0003 (10) | 0.25 |
| Cr | 0.0201±0.0031 (9) | 0.0165±0.0015 (10) | 0.30 |
| Ni | 0.0710±0.0051 (9) | 0.0721±0.0097 (10) | 0.92 |
| Cu | 5.3±0.4 (9) | 5.34±0.53 (10) | 0.93 |
| As | 0.0007±0.0000 (9) | 0.0008±0.0002 (10) | 0.92 |
| Se | 1.15±0.08 (9) | 1.10±0.06 (10) | 0.63 |
| Mo | 0.80±0.04 (9) | 0.77±0.05 (10) | 0.74 |
| Ag | N.D. | N.D. | N.D. |
| Sb | 0.0009±0.0007 (9) | 0.0002±0.0000 (10) | 0.26 |
| Tl | 0.0004±0.0000 (9) | 0.0004±0.0000 (10) | 0.90 |
| Pb | 0.0100±0.0006 (9) | 0.0106±0.0012 (10) | 0.70 |
|  | Kidney | |  |
| Metal ions (ng/mg) | WT | ZIP8KI | P value |
| Be | N.D. | N.D. | N.D. |
| Al | 0.49±0.08 (9) | 0.54±0.16 (10) | 0.81 |
| V | 0.0068±0.0009 (9) | 0.0051±0.0006 (10) | 0.13 |
| Cr | 0.0217±0.0015 (9) | 0.0301±0.0063 (10) | 0.23 |
| Ni | 0.1076±0.0059 (9) | 0.1187±0.0109 (10) | 0.40 |
| Cu | 4.72±0.13 (9) | 4.46±0.14 (10) | 0.18 |
| As | 0.0010±0.0002 (9) | 0.0014±0.0002 (10) | 0.13 |
| Se | 1.39±0.05 (9) | 1.30±0.08 (10) | 0.37 |
| Mo | 0.34±0.01 (9) | 0.33±0.01 (10) | 0.37 |
| Sb | 0.0002±0.0000 (9) | 0.0002±0.0000 (10) | 0.39 |
| Tl | 0.0079±0.0005 (9) | 0.0071±0.0005 (10) | 0.27 |
| Pb | 0.0169±0.0011 (9) | 0.0192±0.0017 (10) | 0.29 |
