# Supplementary figures and images for: The Allelic Variant A391T of Metal Ion Transporter ZIP8 (SLC39A8) Leads to Hypotension and Enhanced Insulin Resistance
Source: Front Physiol. 2022 Jun 15;13:912277. doi: 10.3389/fphys.2022.912277 (PMC9240775; doi:10.3389/fphys.2022.912277)

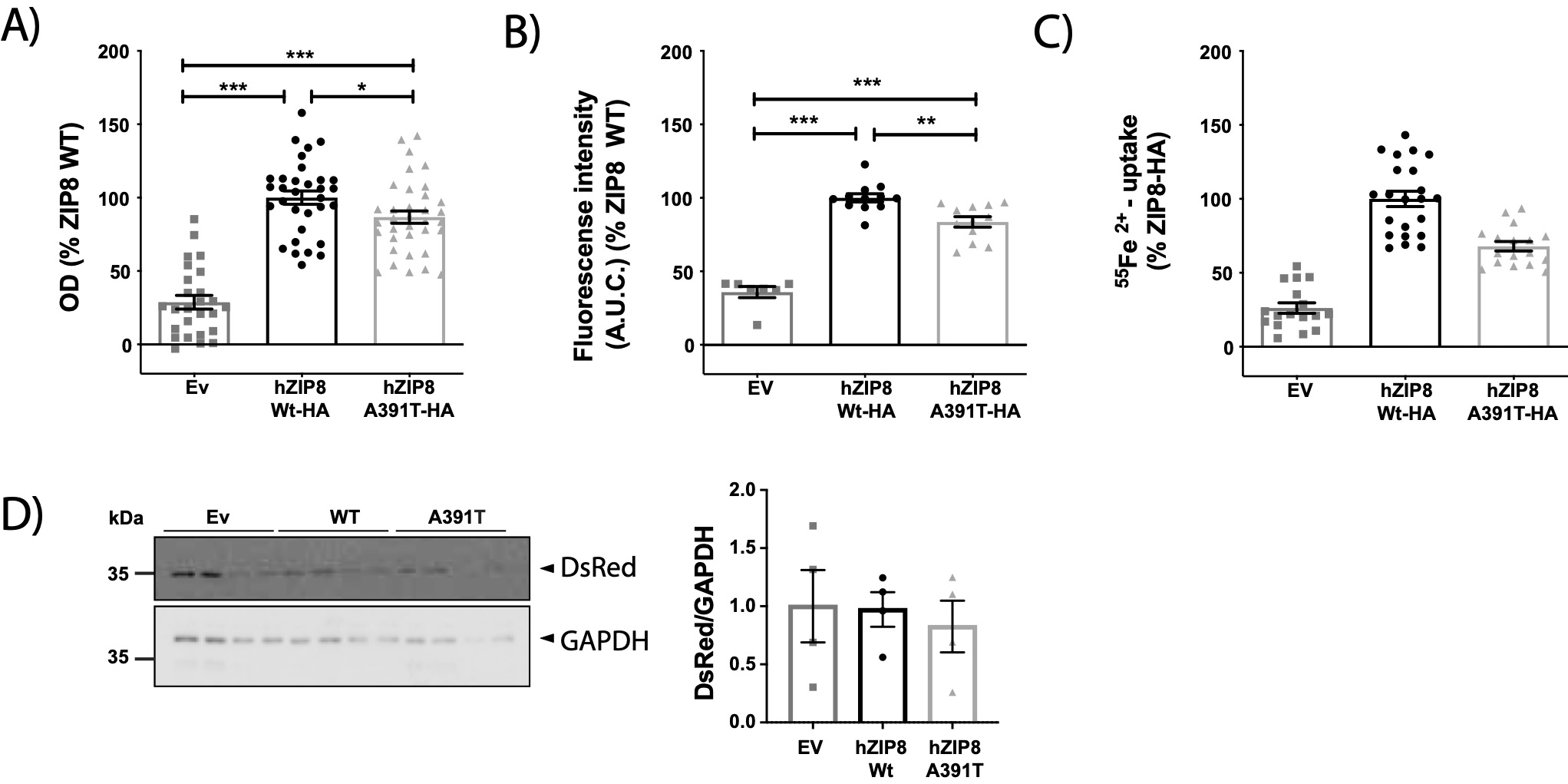

Supplement: Supplementary file 2 [file Image1.JPEG]

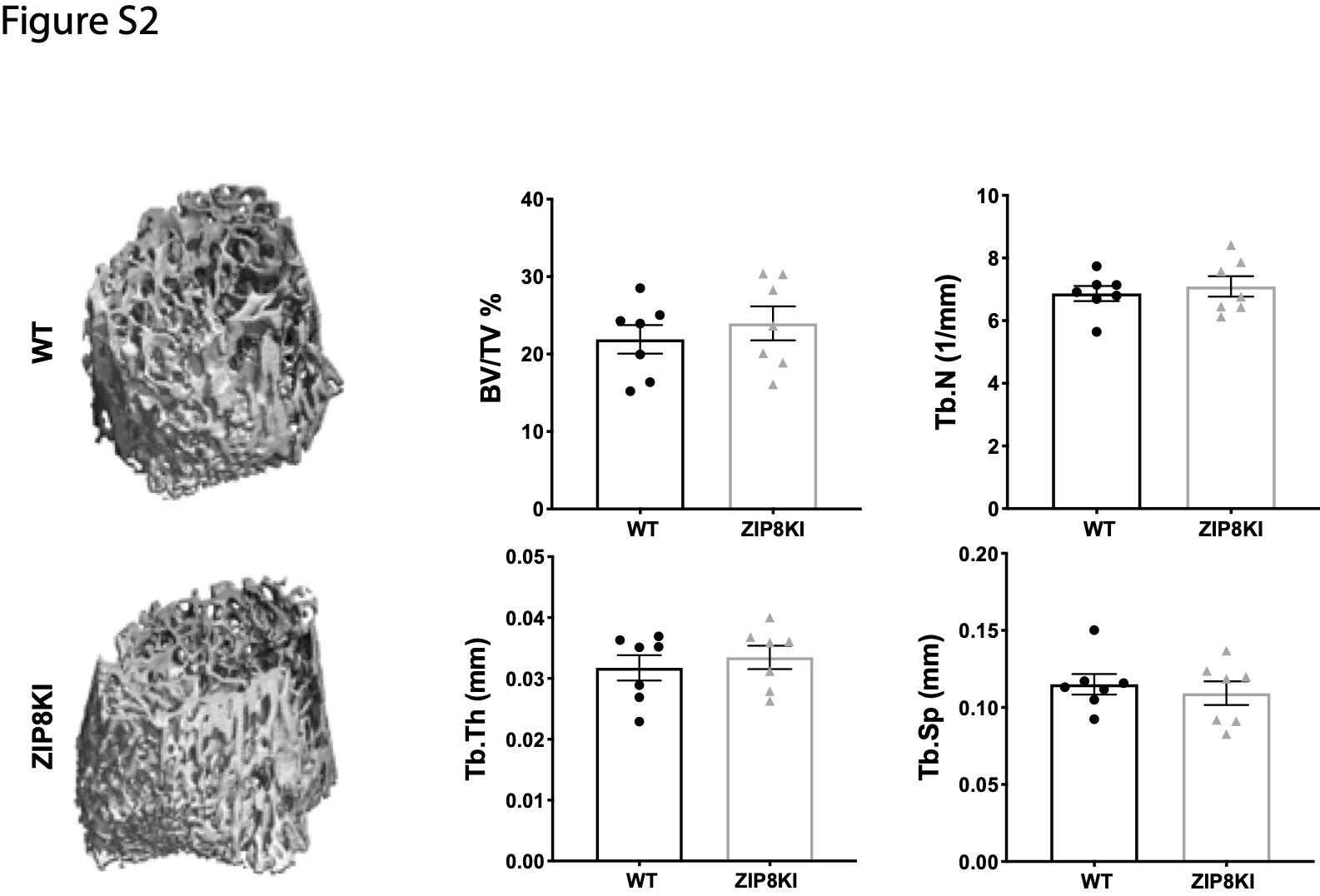

Supplement: Supplementary file 3 [file Image2.JPEG]
