# Supplementary material for: The Allelic Variant A391T of Metal Ion Transporter ZIP8 (SLC39A8) Leads to Hypotension and Enhanced Insulin Resistance
Source: Front Physiol. 2022 Jun 15;13:912277. doi: 10.3389/fphys.2022.912277 (PMC9240775; doi:10.3389/fphys.2022.912277)
Supplement: Supplementary file 4 [file Table2.docx]

## **Supplementary Table 2**

**Supplementary Table 2** Water intake, urine volume and organs weight of male WT and ZIP8KI mice fed with StD. Data shown are means±SEM (n).

|  |  | |  |
| --- | --- | --- | --- |
| Parameter | WT | ZIP8KI | P value |
| Body Weight (g) | 26.5±1.3 (6) | 25.5±0.5 (6) | 0.51 |
| Food consumption/BW (ml/g) | 0.18±0.01 (8) | 0.16±0.02 (7) | 0.56 |
| Water intake/BW (ml/g/24h) | 0.18±0.020(8) | 0.16±0.021(7) | 0.53 |
| Urine volume/BW (ml/g/24h) | 0.060±0.007(8) | 0.065±0.009(7) | 0.68 |
| **Kidney weight/BW** (mg/g) | 5.65±0.17(8) | 6.71±0.43(7) | 0.06 |
| **Liver weight/BW** (mg/g) | 47.5±3.2(8) | 44.34±2.5(7) | 0.46 |
| **Heart weight/BW** (mg/g) | 5.34±0.40(8) | 6.19±0.51(7) | 0.40 |
